# Thiol-based redox signaling in the nitrogen-fixing symbiosis

**DOI:** 10.3389/fpls.2013.00376

**Published:** 2013-09-26

**Authors:** Pierre Frendo, Manuel A. Matamoros, Geneviève Alloing, Manuel Becana

**Affiliations:** ^1^Institut Sophia Agrobiotech, Université de Nice-Sophia AntipolisNice, France; ^2^Institut Sophia Agrobiotech, Institut National de la Recherche Agronomique, Unité Mixte de Recherches 1355Nice, France; ^3^Institut Sophia Agrobiotech, Centre National de la Recherche Scientifique, Unité Mixte de Recherches 7254Nice, France; ^4^Estación Experimental de Aula Dei, Consejo Superior de Investigaciones CientíficasZaragoza, Spain

**Keywords:** (homo)glutathione, legume nodules, reactive nitrogen species, reactive oxygen species, redox regulation, symbiosis

## Abstract

In nitrogen poor soils legumes establish a symbiotic interaction with rhizobia that results in the formation of root nodules. These are unique plant organs where bacteria differentiate into bacteroids, which express the nitrogenase enzyme complex that reduces atmospheric N _2_ to ammonia. Nodule metabolism requires a tight control of the concentrations of reactive oxygen and nitrogen species (RONS) so that they can perform useful signaling roles while avoiding nitro-oxidative damage. In nodules a thiol-dependent regulatory network that senses, transmits and responds to redox changes is starting to be elucidated. A combination of enzymatic, immunological, pharmacological and molecular analyses has allowed us to conclude that glutathione and its legume-specific homolog, homoglutathione, are abundant in meristematic and infected cells, that their spatio-temporally distribution is correlated with the corresponding (homo)glutathione synthetase activities, and that they are crucial for nodule development and function. Glutathione is at high concentrations in the bacteroids and at moderate amounts in the mitochondria, cytosol and nuclei. Less information is available on other components of the network. The expression of multiple isoforms of glutathione peroxidases, peroxiredoxins, thioredoxins, glutaredoxins and NADPH-thioredoxin reductases has been detected in nodule cells using antibodies and proteomics. Peroxiredoxins and thioredoxins are essential to regulate and in some cases to detoxify RONS in nodules. Further research is necessary to clarify the regulation of the expression and activity of thiol redox-active proteins in response to abiotic, biotic and developmental cues, their interactions with downstream targets by disulfide-exchange reactions, and their participation in signaling cascades. The availability of mutants and transgenic lines will be crucial to facilitate systematic investigations into the function of the various proteins in the legume-rhizobial symbiosis.

## SYMBIOTIC NITROGEN FIXATION AND ANTIOXIDANT DEFENSES

Legumes are unique among crop plants in their ability to establish symbiotic associations with soil bacteria known collectively as rhizobia. As a result of a molecular dialog between the legume cells and rhizobia, nodules are formed on the roots or, in a few cases, on the stems. Nodules are organs specialized in dinitrogen (N_2_) fixation, a biological process in which atmospheric dinitrogen is reduced to ammonia by the nitrogenase enzyme complex of the bacteroids (for a review, see Vance, 2008). The energy required for N_2_ fixation derives ultimately from sucrose transported from the leaves to the nodules. In return, the ammonia produced by the bacteroids is assimilated into organic compounds to fulfill the nitrogen demand of both the bacteria and the plant.

Nodules contain abundant metalloproteins, including leghemoglobin and nitrogenase, which are prone to oxidation generating reactive oxygen and nitrogen species (RONS). Some RONS, such as the superoxide radical ($O_{2}^{•}$), hydrogen peroxide (H_2_O_2_) and nitric oxide (NO), perform signaling functions and have been detected in nodules using cytochemical staining, specific fluorescent probes or electron paramagnetic resonance (Santos et al., 2001; Rubio et al., 2004; Sánchez et al., 2010; del Giudice et al., 2011). However, these RONS are potentially cytotoxic, giving rise to highly oxidizing hydroxyl radicals (^•^OH), nitrogen dioxide (NO_2_) and peroxynitrite (ONOO^-^) if their concentrations are not tightly controlled by antioxidant enzymes and metabolites. Nodule antioxidants include ascorbate, thiol tripeptides, superoxide dismutases, catalases, thiol peroxidases and the enzymes of the ascorbate-glutathione pathway (Becana et al., 2010). Here, we will focus on those antioxidants of nodules whose protective and regulatory functions entail thiol groups, paying special attention to the contribution of their redox activities to the lifespan of the symbiosis, from root cell infection to nodule senescence.

## BIOSYNTHESIS OF THIOL TRIPEPTIDES IN LEGUMES

### THIOL COMPOUNDS AND THE THIOL BIOSYNTHETIC PATHWAY

The thiol tripeptide glutathione (GSH; γGlu-Cys-Gly) and ascorbate are the major water-soluble antioxidants and redox buffers of plants. In addition, GSH performs multiple and diverse functions, including regulation of cell cycle, sulfur transport and storage, stress responses and detoxification of heavy metals and xenobiotics (Rausch et al., 2007; Foyer and Noctor, 2011). In legumes, the structural homolog, homoglutathione (hGSH; γGlu-Cys-βAla), may partially or completely replace GSH (Frendo et al., 2001; Matamoros et al., 2003). Both compounds can be found at concentrations of 0.5–1.5 mM in nodules (Matamoros et al., 1999b), similar to the estimated ranges of 1–3 mM GSH and 0.4–0.7 mM hGSH in the chloroplasts (Bergmann and Rennenberg, 1993) or 2–3 mM GSH in the cytosol of root cells (Fricker et al., 2000).

**FIGURE 1:**
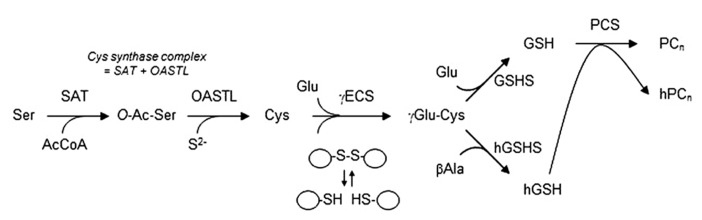
**Schematics of the (h)GSH biosynthetic pathway.** Depicted are also the enzymes forming the cysteine synthase complex, namely, serine acetyltransferase (SAT) and *O-*acetylserine(thiol)lyase (OASTL), as well as those involved in phytochelatin (PC_n_) and homophytochelatin (hPC_n_) synthesis. For the γECS enzyme, the redox switch is drawn as an equilibrium between the more active (oxidized) dimeric form and the less active (reduced) monomeric form. Other abbreviations not used in the text: *O*-Ac-Ser, *O*-acetylserine; γGlu-Cys, γ-glutamylcysteine.

The synthesis of GSH in plants and other organisms is accomplished in two sequential reactions (**Figure 1**) catalyzed by γ-glutamylcysteine synthetase (γECS) and GSH synthetase (GSHS), both showing a strict requirement for ATP and Mg^2^^+^ (Bergmann and Rennenberg, 1993). In legumes, the synthesis of hGSH is also carried out in two steps, involving the same γECS enzyme and a specific hGSH synthetase (hGSHS), which exhibits a much higher affinity for β-alanine than for glycine (Macnicol, 1987; Klapheck et al., 1988; Frendo et al., 2001; Iturbe-Ormaetxe et al., 2002). Detailed work using site-directed mutagenesis of hGSHS has conclusively shown that only two contiguous amino acid residues in the active site (Leu-534 and Pro-535 in *Medicago truncatula* hGSHS, and Leu-487 and Pro-488 in soybean (*Glycine max*) hGSHS) determine the substrate preference for β-alanine over glycine (Frendo et al., 2001; Galant et al., 2011).

The *GSHS* and *hGSHS* genes share high homology (~70% amino acid identity) and are located in tandem on the same chromosome in the model legumes *M. truncatula* (Frendo et al., 2001) and *Lotus japonicus* (Matamoros et al., 2003). These findings are consistent with the proposal that the *hGSHS* gene derives from the *GSHS* gene by a duplication event occurred after the divergence between the Fabales, Solanales and Brassicales (Frendo et al., 2001). Despite this close relationship, the two genes are differentially regulated in plant organs and in response to stressful conditions or signal compounds such as hormones and RONS. This can be examplified with studies performed on the two model legumes. Thus, *M. truncatula* produces exclusively GSH in the leaves and both GSH and hGSH in the roots and nodules (Frendo et al., 1999), whereas *L. japonicus* produces almost exclusively hGSH in the roots and leaves, but more GSH than hGSH in the nodules (Matamoros et al., 2003). In legumes, the thiol contents are positively correlated with the GSHS and hGSHS activities and in general with their mRNA levels (Frendo et al., 1999; Matamoros et al., 1999b, 2003). In *M. truncatula* roots, the expression of *γECS* and *GSHS* but not of *hGSHS* is induced by NO (Innocenti et al., 2007). In *L. japonicus* roots, NO, cytokinins and polyamines up-regulated *GSHS* but not *hGSHS*, whereas hGSHS mRNA and activity were induced by auxins (Clemente et al., 2012). Taken together, these observations suggest the presence of gene-specific *cis-*acting regulatory elements in the *GSHS* and *hGSHS* promoters. However, despite the long time elapsed since the discovery of hGSH in legumes, the reason why this thiol replaces GSH in some legume species and tissues is still a mistery.

Other thiol compounds can be found in plants, but little is known about their roles in the symbiosis and hence they will be only very briefly described here. The GSH and hGSH precursors, cysteine and γ-glutamylcysteine, are found in nodules at concentrations considerably lower (<15%) than GSH or hGSH, in the range of 30–120 μM. As occurs for the tripeptides, both precursors are more abundant in nodules than in roots and leaves, pointing out an active thiol metabolism in N_2_-fixing nodules (Matamoros et al., 1999b). This conclusion is reinforced by the high transcript levels of the two enzymes involved in cysteine synthesis, serine acetyltransferase and *O*-acetylserine(thiol)lyase (**Figure 1**), that can be found in nodules (*M. truncatula* Gene Expression Atlas^1^ and *L. japonicus* Gene Expression Atlas^2^). Another thiols that can be found in legume nodules and other plant organs are (homo)phytochelatins. These are cysteine-rich polypeptides of general structure (γGlu-Cys)_2__-__11_-Gly or (γGlu-Cys)_2__-__11_-βAla, which are synthesized from (h)GSH only in the presence of certain metals and metalloids, such as selenium, cadmium, mercury or lead (**Figure 1**). It has been shown that these polypeptides form complexes with cadmium, which is then sequestrated to the vacuoles, avoiding poisoning of cellular metabolism. Interestingly, three functional phytochelatin synthase genes were found in *L. japonicus*, which differ in their cadmium response and are all expressed in nodules (Ramos et al., 2007).

### REGULATION AND LOCALIZATION OF THIOL SYNTHESIS

The *γECS*, *GSHS *and *hGSHS* genes can be transcriptionally regulated in response to RONS and hormones, as mentioned above. A notable case of this type of regulation is the coordinated induction of the *γECS* and *GSHS *genes of *Arabidopsis thaliana* (Xiang and Oliver, 1998) and of the three genes of *L. japonicus* (Clemente et al., 2012) exposed to jasmonic acid. However, it has also been demonstrated that the (h)GSH biosynthetic pathway can be controlled at the translational and post-translational levels by modulation of the γECS mRNA stability and enzyme activity, respectively (Rausch et al., 2007; Galant et al., 2011). The post-translational regulation of plant γECS enzymes would occur *via* a conserved intermolecular disulfide bond that is likely to operate *in vivo* as a redox switch, in such a way that oxidation shifts the equilibrium toward the more active, dimeric form (Galant et al., 2011; **Figure 1**).

An additional, but by no means less important, mechanism of regulation may rest on the compartmentation of the thiol biosynthetic pathway. In nodules, subcellular fractionation and immunogold labeling studies have shown that γECS is localized in plastids, whereas GSHS and hGSHS are localized in both the plastids and cytosol (Moran et al., 2000; Clemente et al., 2012). A localization of GSHS in cowpea (*Vigna unguiculata*) nodule mitochondria needs to be confirmed by electron microscopy and examined in other legume nodules (Moran et al., 2000). Similar subcellular localizations have been reported for the enzymes of *A. thaliana*, where γECS is confined to the plastids and GSHS is predominantly located to the cytosol (Rausch et al., 2007; Galant et al., 2011). Because γ-glutamylcysteine needs to be exported from the plastids to the cytosol, where most (h)GSH synthesis takes place, subcellular compartmentation provides a potential conduit for transmitting redox signals out of the chloroplasts and probably of other plastids (Mullineaux and Rausch, 2005).

## ROLES OF THIOLS IN NODULE FORMATION AND FUNCTIONING

A recent electron microscopy study of pea (*Pisum sativum*) nodules with a GSH-specific antibody revealed that this thiol is present in the bacteroids, mitochondria, cytosol and nuclei of infected cells (Matamoros et al., 2013). Furthermore, as nodules progress from the young to mature stage, total glutathione (reduced + oxidized) decreases in the mitochondria but increases in the bacteroids, cytosol and nuclei, which indicates differential turnover of the thiol or its redistribution between nodule compartments. The finding of GSH in nuclei of infected cells suggests that the thiol performs additional functions to the regulation of the cell cycle, which will be more important in meristematic cells (Diaz Vivancos et al., 2010). These functions may include DNA antioxidative protection or redox regulation of transcription factors (Matamoros et al., 2013).

At the tissue level, careful dissection of nodules has shown that, in general, the (h)GSH content and the γECS, GSHS and hGSHS activities are particularly high in the meristematic and infected zones of legume nodules (Matamoros et al., 1999b). Remarkably, hGSHS is very active in the cortex of bean nodules. The reasons of this specific distribution are unknown, but could be related to a function of this protein in the vascular bundles or in the O_2_ diffusion barrier, which are localized to the nodule cortex. These observations have been recently corroborated by using promoter-GUS fusions. Thus, El Msehli et al. (2011) have determined the spatio-temporal gene expression of the (h)GSH synthesis pathway in *M. truncatula*. The expression of *γECS* appears to be higher in the meristematic and infection zones of nodules, whereas the *hGSHS* mRNA is more abundant in the cortex and the *GSHS* mRNA in the cortex and in the N_2_-fixing zone.

The concentration of (h)GSH and the N_2_-fixing activity of nodules are positively correlated during nodule development (Dalton et al., 1993). The two parameters decline with advancing age (Evans et al., 1999; Groten et al., 2005) as well as during stress-induced senescence (Escuredo et al., 1996; Gogorcena et al., 1997; Matamoros et al., 1999a; Marino et al., 2007; Naya et al., 2007). These findings suggest that (h)GSH is important for nodule activity, a hypothesis that was tested by modulating the nodule content of (h)GSH by pharmacological and genetic approaches. The application of a specific inhibitor of (h)GSH biosynthesis (buthionine sulfoximine) or the expression of *(h)GSHS* in antisense orientation caused depletion of (h)GSH in *M. truncatula* roots (Frendo et al., 2005). The deficiency of (h)GSH synthesis in roots decreased substantially the number of nascent nodules and the expression of some early nodulin genes (Frendo et al., 2005). These results, along with the proposed role of GSH in meristem formation in *A. thaliana* (Vernoux et al., 2000; Reichheld et al., 2007), suggest that (h)GSH is required for the initiation and maintenance of the nodule meristem. The transcriptomic analysis of (h)GSH-depleted plants during early nodulation revealed down-regulation of genes implicated in meristem formation and up-regulation of salicylic acid-related genes after infection with *Sinorhizobium meliloti* (Pucciariello et al., 2009). The potentially enhanced expression of defense genes provides a partial explanation for the negative effects of (h)GSH depletion on the symbiosis. Likewise, the reduction of (h)GSH content in transgenic roots led to a significantly lower N_2_-fixing activity, which was related to a smaller nodule size (El Msehli et al., 2011). Conversely, the overexpression of γECS resulted in an elevated (h)GSH content, which was associated with enhanced N_2_ fixation. All these data underpin the importance of (h)GSH in nodule development and functioning.

Although the precise roles of (h)GSH in the onset and life-span of symbiosis are still to be defined, a central role of (h)GSH in the regulation of symbiotic activity *via* hormone transduction pathways can be already anticipated (Bashandy et al., 2010; Clemente et al., 2012). Moreover, GSH and hGSH act as substrates for key antioxidant enzymes, such as glutathione reductases, glutathione-*S*-transferases and glutaredoxins (Grxs), and hence both thiols probably participate in the regulation of symbiosis* via* modulation of enzyme activities (Dalton et al., 2009).

## THIOL PEROXIDASES AND OTHER REDOXINS OF NODULES

**FIGURE 2:**
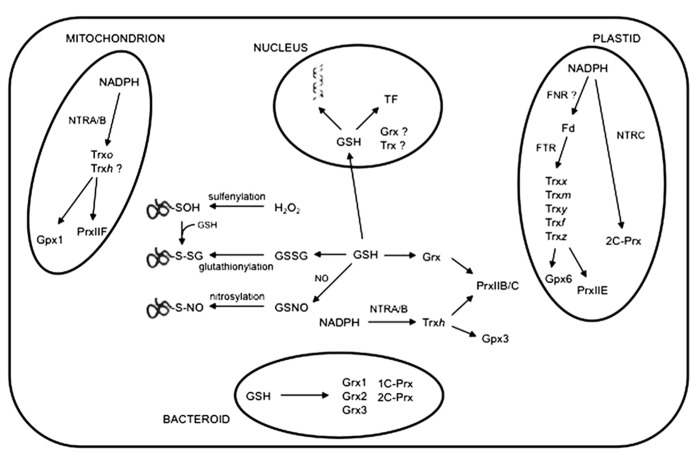
**A simplified overview of the thiol-based redox network in legume nodules.** Representative cellular compartments of an infected cell showing their contents of proteins, such as Gpxs, Prxs and other redoxins, that contain active cysteine residues. The figure includes also the NTRA/B-Trx-Prx redox systems in the mitochondria and cytosol, and the NTRC-Prx and FTR-Trx redox systems in the plastids. Post-translational modifications involving protein cysteine residues, such as sulfenylation (oxidation of the cysteine thiolate to sulfenic acid), glutathionylation (incorporation of a glutathione moiety) and nitrosylation (incorporation of a nitrosyl group), are shown. GSH in the nucleus may protect DNA from oxidative damage by RONS and modulate activity of transcription factors (TF), activating or inactivating defense and stress-related genes. For simplicity, GSH in the mitochondria or plastids is not indicated. Other abbreviations not used in the text: Fd, ferredoxin; FNR, ferredoxin-NADP^+^ reductase; FTR, ferredoxin-thioredoxin reductase.

Glutathione peroxidases (Gpxs), peroxiredoxins (Prxs) and thioredoxins (Trxs) are protein components of a regulatory network system (**Figure 2**) that perceives, modulates and transmits information of the cellular redox state *via* thiol-disufide exchange (Dietz et al., 2006; Meyer et al., 2009). Although phylogenetically distant, plant Gpxs and Prxs catalyze similar biochemical reactions (Rouhier and Jacquot, 2005). During their catalytic mechanism, both types of thiol peroxidases reduce H_2_O_2_, lipid hydroperoxides or, in some cases, ONOO^-^, with formation of a sulfenic acid (–SOH) or a disulfide bond on the proteins. Cysteine thiol groups are regenerated by Trxs, which are in turn reduced by NADPH-thioredoxin reductases (NTRs) with the consumption of NADPH. It has been also shown that Grxs can substitute for Trxs as electron donors of some Prxs (Rouhier and Jacquot, 2005). Oxidized Grxs are reduced by GSH and glutathione reductase using the reducing power of NADPH.

Thiol peroxidases are widely distributed in all organisms and are encoded by small multigenic families. The *A. thaliana* and poplar (*Populus trichocarpa*) genomes contain eight and six Gpxs, respectively, that are differentially regulated at the transcriptional level in plant organs and in response to stress conditions and growth regulators (Rodriguez Milla et al., 2003; Navrot et al., 2006). The *L. japonicus *genome contains six *Gpx* genes encoding cytosolic, plastidial and mitochondrial isoforms (Ramos et al., 2009). In nodules, the *Gpx3* gene showed a remarkable up-regulation relative to uninfected roots (Colebatch et al., 2002; Ramos et al., 2009). Interestingly, prediction programs of subcellular localization suggest that Gpx3 is targeted to the secretory pathway and/or cytosol and could thus participate in the perception and transduction of redox changes in the nodule apoplast *via* thiol-disulfide exchange reactions with membrane proteins. An unequivocal localization of Gpx3 is necessary to clarify its role in nodule cells. In a recent immunolocalization study, Gpxs were found in the amyloplasts of nodules of *L. japonicus*, alfalfa (*Medicago* sativa) and *Sesbania rostrata*. In most cases, labeling was associated to starch grains, which hints to a role of Gpxs in the regulation of starch metabolism (Ramos et al., 2009).

In plants, Prxs are grouped into four classes (PrxQ, PrxII, 2-CPrx and 1-CPrx) that differ in their catalytic mechanisms and subcellular locations (Dietz et al., 2006). The *L. japonicus* genome encodes eight Prxs, which are localized to the chloroplasts (PrxQ, 2C-PrxA, 2C-PrxB and PrxIIE), mitochondria (PrxIIF), cytosol (PrxIIB/C) and nucleus (1C-Prx). These proteins show specific organ distribution. Thus, 1C-Prx localizes mainly to the embryo and PrxQ levels are very high in leaves compared to other organs. Nodules contain PrxIIB/C in the cytosol, PrxIIF in the mitochondria and low levels of 2C-Prx and PrxIIE in the plastids (Tovar-Méndez et al., 2011). These proteins are part of Prx-Trx-NTR systems that are operative in the cytosol, plastids and mitochondria (**Figure 2**). Trxs form a complex family of disulfide oxidoreductases involved in the redox regulation of cell metabolism. In plant tissues, several groups of Trxs have been identified. Trx*f*, Trx*m*, Trx*x*, Trx*y* and Trx*z* are localized in the plastids, Trx*h* in the cytosol and Trx*o* in the mitochondria. In addition, some Trx*h* isoforms have been found in the mitochondria, nucleus and endoplasmic reticulum (Meyer et al., 2009). In legumes, the Trx protein family has been analyzed in detail in *M. truncatula* (Alkhalfioui et al., 2008) and *L. japonicus* (Tovar-Méndez et al., 2011). In nodules, the cytosolic Trx*h* mRNAs are very abundant, whereas mitochondrial Trx*o* is moderately expressed and plastidial Trx mRNAs are poorly represented. The Trx*h* and Trx*o* isoforms are maintained in reduced form by cytosolic and mitochondrial NTRs, whereas in the plastids this function is probably performed by the sequential action of ferredoxin-NADP and ferredoxin-Trx reductases (Tovar-Méndez et al., 2011). Nodule plastids also contain low levels of a singular NTR protein, NTRC, characterized by the presence of a Trx domain that enables the enzyme to reduce directly 2C-Prxs with high catalytic efficiency (Pulido et al., 2010). On the other hand, *M. truncatula* contains two novel Trx isoforms, Trx*s1* and Trx*s2*, which are associated with symbiosis (Alkhalfioui et al., 2008). The function of these proteins in the nodules awaits clarification. Notably, no orthologs were found in *A. thaliana*, *L. japonicus*, soybean and pea, suggesting that the Trx*s* isoforms could be unique to certain legume species. The information on Grxs in legume nodules is still very scarce. Proteomic analyses identified two Grxs (GrxC2 and GrxC4) in *L. japonicus* nodules (Tovar-Méndez et al., 2011). As in other plant organs, nodule Grxs probably constitute an alternative reducing system to Trxs (**Figure 2**). Moreover, biochemical studies suggest that Grxs could perform specific functions, such as deglutathionylation, more efficiently than Trxs (Meyer et al., 2009).

The presence of multiple isoforms of thiol peroxidases, Trxs and Grxs in cell compartments and plant organs reflects the necessity of a tight thiol-based control of redox homeostasis for plant function. Furthermore, these proteins probably fulfill redox-independent specific roles due to their differential ability to interact with other proteins. The crucial role of Trxs in nodules was demonstrated by the finding that soybean hairy roots with RNAi-suppressed levels of a Trx*h* isoform showed a severe impairment of nodule formation and development (Lee et al., 2005). Nevertheless, the precise functions of the thiol-based regulatory network in the N_2_-fixing symbiosis remain largely undefined.

Early work has established that Gpxs, Prxs and Trxs are involved in the plant’s response to environmental constraints (Dietz et al., 2006; Navrot et al., 2006; Meyer et al., 2009). However, direct evidence for a similar function in legume nodules is still lacking. The thiol redox system has been recently investigated during the natural senescence of common bean (*Phaseolus vulgaris*) nodules (Matamoros et al., 2013). Contrary to cytosolic PrxIIB/C, the content of mitochondrial PrxIIF remains constant in aging nodules (Groten et al., 2006; Matamoros et al., 2013). The mitochondrial redox status influences nuclear gene expression and cell fate *via* retrograde signaling (Rhoads and Subbaiah, 2007), and PrxIIF might participate in this process. On the other hand, nodule mitochondria are an early target of oxidative modifications in aging nodules and have increased levels of oxidized lipids and proteins (Matamoros et al., 2013). PrxIIF may protect mitochondria from excessive oxidative damage and thus warrant mitochondrial activity late in nodule development.

## THIOL-BASED REDOX REGULATORY NETWORK IN BACTEROIDS

The intracellular redox state of the bacterial partner appears to play an important signaling role in the establishment and functioning of symbiosis. Bacteroids are endowed with multiple antioxidant enzymes, including peroxidases, catalase, Prxs and Grxs, to modulate or detoxify RONS (**Figure 2**). Alteration of RONS levels in mutant strains have profound effects at different stages of symbiosis (Puppo et al., 2013). We will briefly refer to some recent advances concerning thiol-dependent redox signaling in bacteroids.

### THE IMPORTANCE OF BACTERIAL GLUTATHIONE DURING SYMBIOSIS

The first direct evidence that the GSH pathway of the bacterial symbiotic partner is important for nodulation and N_2_ fixation was obtained by using *S. meliloti* mutants impaired in GSH synthesis. In *S. meliloti*, as in other organisms, GSH is synthesized by γECS and GSHS, encoded, respectively, by the *gshA* and *gshB* genes. An *S. meliloti* mutant deficient in *gshA* was unable to grow under non-stress conditions, precluding any nodulation on alfalfa. Conversely, a *gshB* mutant was able to grow and nodulate alfalfa, indicating that γ-glutamylcysteine can partially compensate for GSH deficiency. The *gshB* strain showed nevertheless a delayed-nodulation phenotype coupled to abnormal development and early senescence of nodules. Both *gshA* and *gshB* mutants exhibited higher catalase activity than the wild-type, suggesting that the two mutants were experiencing oxidative stress (Harrison et al., 2005). Furthermore, *gshB* mutants of* Rhizobium tropici* and *Rhizobium etli *were affected in their ability to compete during nodulation of common bean, and nodules induced by *gshB* mutants displayed early senescence (Riccillo et al., 2000; Tate et al., 2012). A deficiency in GSH was associated with increased levels of $O_{2}^{•}$ radicals in nodules infected with the *gshB* mutant of *R. tropici*, and thus antioxidant mechanisms dependent on bacterial GSH might be impaired (Muglia et al., 2008). In *R. etli*, GSH deficiency was linked to a reduction of glutamine uptake in growing cultures, suggesting a complex GSH-glutamine metabolic relationship that may be important for symbiotic efficiency (Tate et al., 2012). Finally, the mutation in the *gshA* gene of *Bradyrhizobium* sp. 6144-S7Z appears to affect the ability of the bacterium to compete during peanut (*Arachis hypogaea*) nodulation, but not its capacity to form effective nodules (Sobrevals et al., 2006). Taken together, these results show that the bacterial GSH pool plays a critical role in the rhizobia-plant interaction and that different cellular processes are regulated by, or are dependent on, GSH in free-living rhizobia and in N_2_-fixing bacteroids.

### THE ROLE OF BACTERIAL GRX AND TRX PATHWAYS

An* in silico* analysis of the *S. meliloti* genome led to the identification of three genes that putatively encode Grxs from different classes (**Figure 2**): Grx1, containing the dithiol CGYC active site of class I Grxs; Grx2, containing the monothiol CGFS active site of class II Grxs; and Grx3, carrying two domains, an N-terminal Grx domain with a CPYG active site and a C-terminal domain with a methylamine utilization protein motif (Benyamina et al., 2013). Inactivation of one gene or the other showed that Grx1 and Grx2 play different roles, Grx1 in protein deglutathionylation and Grx2 in regulation of iron metabolism. Both *grx1* and *grx2* mutants were impaired in bacterial growth and in nodule functioning. On one hand, *grx1 *inactivation led to nodule abortion and absence of bacteroid differentiation; on the other, *grx2 *inactivation decreased nodule development without modifying bacteroid differentiation. Therefore, both Grx1 and Grx2 appear to be critical proteins for optimal development of the N_2_-fixing symbiosis. The incapacity of the *grx1 *mutant to differentiate is remarkably similar to the phenotype of a mutant (*katB katC*) affected in catalase activity (Jamet et al., 2003). This observation emphasizes the importance of a fine tuning of the RONS balance in bacteroid differentiation and the key role of *S*-glutathionylation in modulating the function of proteins essential for this process.

In *Escherichia coli*, the Grx and Trx pathways, the two branches of the thiol-redox system, are functionally redundant. Whereas the simultaneous inactivation of the two pathways is non-viable, inactivation of either of them is viable, indicating that each pathway can fully carry out the essential function of reducing disulfides in the absence of the other (Toledano et al., 2007). This does not appear to be the case in *S. meliloti*, where *grx1* and *grx2* mutants were affected in both free-living bacteria and plant-hosted bacteroids, which points out that Grx1 and Grx2 have more specific roles than the corresponding *E. coli* enzymes. Consistent with the notion of poor redundancy, Trx-like proteins are required for optimal N_2_-fixation efficiency in *S. meliloti *and *Rhizobium leguminosarum *(Vargas et al., 1994; Castro-Sowinski et al., 2007).

## POST-TRANSLATIONAL MODIFICATIONS AND REDOX SIGNALING IN NODULES

Several lines of evidence indicate that RONS are key signals that regulate the establishment of symbiosis (Ramu et al., 2002; del Giudice et al., 2011; Puppo et al., 2013). Differences in the intensity, duration and localization of RONS might be perceived and transmitted by thiol-dependent mechanisms. Transcription factors that respond to redox changes by interaction with Trxs have been described in yeast, plants and animals (Buchanan and Balmer, 2005). In legumes, the expression of several transcription factors involved in the onset of symbiosis seems to be regulated by RONS (Andrio et al., 2013). Moreover, NO is required for the transcriptional control of genes involved in nodule development (del Giudice et al., 2011). Consequently, it is expected that the thiol-based regulatory network plays relevant functions in the transcriptional reprogramming that takes place during the initial stages of the symbiotic interaction.

The important signaling role of NO can be modulated by the reaction of this short-lived free radical with GSH and presumably hGSH, in the presence of O_2_, to form the corresponding *S*-nitrosothiols. In particular, nitrosoglutathione (GSNO) is considered a carrier and reservoir of NO in plant cells, where it participates in transnitrosylation reactions, delivering NO to protein cysteine residues (Corpas et al., 2011). In turn, the bioactivity of GSNO can be regulated by the enzyme *S*-nitrosoglutathione reductase (GSNOR), also termed class III alcohol dehydrogenase, which catalyzes the NADH-dependent reduction of GSNO to glutathione disulfide and ammonia (Corpas et al., 2011). The concentrations of GSNO or nitrosohomoglutathione (hGSNO) have not been determined yet in nodules. However, treatment of *M. truncatula* seedlings with GSNO in hydroponic medium causes a massive induction of genes involved in key processes, such as primary metabolism and defense response, in roots and in young and mature nodules, pointing out that this compound may perform important functions *in vivo* during rhizobial infection and nodule development (Ferrarini et al., 2008). Likewise, the GSNOR transcript is clearly detectable in nodules, roots, leaves and seeds, suggesting that the enzyme is functional in all these plant organs (*M. truncatula* Gene Expression Atlas^1^ and *L. japonicus* Gene Expression Atlas^2^).

The regulation of protein function *via* oxidative modification has emerged as an important molecular mechanism that modulates various biological processes. Protein cysteine residues are sensitive targets of glutathione disulfide and RONS (**Figure 2**), leading to post-translational modifications such as glutathionylation, nitrosylation and sulfenylation (Couturier et al., 2013; Kovacs and Lindermayr, 2013). These cysteine-based modifications regulate protein function, localization and/or turnover (Klomsiri et al., 2011; Spoel and Loake, 2011). Proteomic analysis brought to light a hundred of sulfenylated proteins in the *M. truncatula*- *S. meliloti* symbiosis (Oger et al., 2012). The major functional group of sulfenylated proteins identified at 2 days post-inoculation was represented by redox-active proteins, but this was not the case for mature nodules, in which the main targets of sulfenylation were proteins related to primary metabolism. Thus, RONS-induced modifications of proteins occur during N_2_ fixation and may be involved in the onset and functioning of symbiosis (Oger et al., 2012). Eighty proteins have also been identified in mature nodules by proteomics as targets of *S-*nitrosylation (Puppo et al., 2013). Twenty-seven proteins were found to be sensitive to both sulfenylation and *S*-nitrosylation. As occurs for sulfenylation, a large part of nitrosylated proteins was found to be related to carbon, nitrogen or energy metabolism, which strengthens the potential role of the cysteine redox state of proteins in the regulation of nodule metabolism. In addition, highly oxidizing NO derivatives, such as ONOO^-^, NO_2_ or nitrosonium cation (NO^+^), may also participate in post-translational modifications such as tyrosine and heme nitration, as demonstrated for glutamine synthetase (Melo et al., 2011) and leghemoglobin (Navascués et al., 2012), respectively.

Redox regulation *via* cysteine residues is also important for the bacterial partner of the symbiosis, and this will be briefly illustrated here with a few examples. In *Bradyrhizobium japonicum*, the cellular pool of active FixK2, a crucial regulator of genes required for the micro-oxic lifestyle, is partly controlled at the post-translational level (Mesa et al., 2009). The FixK2 activity is modulated by an oxidative-dependent inactivation involving a critical cysteine residue near the DNA-binding domain. This post-translational modification might be a strategy to prevent the detrimental activation of the FixK2 regulon depending on the cellular status. The expression of the *fixK2* gene itself is activated by the FixLJ system in response to a moderate decrease of O_2_ tension. Particularly, in bacteroids, where ROS are assumed to be generated as a side product of the high respiration turnover, the FixK2 transient inactivation could prevent the generation of more ROS and guarantee an adequate balance between the beneficial and detrimental effects of respiration.

Thiol-dependent redox sensing also modulates the activity of antioxidant enzymes such as Prxs. Interestingly, an atypical 2C-Prx of *R. etli* is involved in the defense of bacteroids against H_2_O_2_ stress and could require the Trx system as a source of reducing power (Dombrecht et al., 2005). An *S. meliloti 1C-Prx* gene is predominantly expressed in alfalfa root nodules and the protein was detected by proteomic analysis (Puppo et al., 2013). Among the twenty sulfenylated enzymes detected in bacteroids, proteins related to carbohydrate and nitrogen metabolism are largely represented, showing that sulfenylation may regulate the activity of crucial proteins for nodule functioning (Oger et al., 2012).

In indeterminate nodules, bacteroid differentiation is mediated by nodule-specific cysteine-rich (NCR) peptides, which are defensin-type antimicrobial peptides (Mergaert et al., 2003; Kereszt et al., 2011). These NCRs are targeted *via* the plant cell secretory pathway to the symbiosomes, where they trigger bacterial differentiation and/or membrane damage and permeabilization (Van de Velde et al., 2010; Wang et al., 2010). Modifying the cysteine residues or the disulfide configuration of an NCR has been shown to influence its activity against *S. meliloti,* suggesting that a tight control of NCR redox status is a prelude to bacteroid terminal differentiation (Haag et al., 2010).

## CONCLUSIONS

Over the last two decades, many advances have been made on the characterization, regulation and localization of the enzymes of the thiol biosynthetic pathway in model and crop plants. Notable accomplishments include the findings that the pathway is compartimentalized in plant cells, with export of γ-glutamylcysteine from the plastids to the cytosol; the crystallization and subsequent structure elucidation of γECS, GSHS and hGSHS; the role of thiol compounds and associated enzymes in redox signaling and in controlling the cell cycle; the transcriptional regulation of γECS, GSHS and hGSHS by RONS and hormones; and the post-translational regulation of γECS by a redox switch involving conserved cysteine residues. Importantly also in the case of legumes, the use of enzyme inhibitors and transgenic plants has demonstrated that thiol tripeptides are essential for the functioning of the rhizobia-legume symbiosis.

Several important questions need, however, to be solved. Further research will be required to establish if GSH and hGSH perform distinct functions, especially in redox homeostasis and signaling during nodule development. Our knowledge on the function of other components of the thiol regulatory network in legume nodules is still at its infancy. This may be due to the amazingly high number of Prx, Gpx, Trx and Grx isoforms, which are present in multiple cellular compartments and differ in biochemical properties. Also, it will be necessary to assess the role of thiol peroxidases and other redoxins during rhizobial infection and to identify their target nodule proteins. Redox-dependent post-translational modifications constitute a versatile adaptive mechanism to changing conditions. The recent development of redox proteomics permits the large-scale identification of proteins that are modified in response to specific stimuli. To shed light on the signaling events that take place in response to RONS, it will be important to characterize nodule proteins that undergo oxidation, nitrosylation or glutathionylation of critical cysteines, and to investigate the impact of these modifications on their biological activities. The generation of mutants and/or transgenic lines will be most helpful to establish the function of individual proteins and metabolites in the rhizobia-legume symbiosis. Finally, a comparative study of the thiol-based signaling mechanisms underpinning the symbiotic and pathogenic interactions and the plant responses to environmental cues will provide critical information to enhance nitrogen nutrition in crop legumes as well as their tolerance to abiotic and biotic stress. The improvement of the N_2_ fixation efficiency is expected, in turn, to have direct beneficial consequences for sustainable agriculture and the environment as this biological process will eventually lead to a reduction in the input of costly and contaminating nitrogen fertilizers.

## Conflict of Interest Statement

The authors declare that the research was conducted in the absence of any commercial or financial relationships that could be construed as a potential conflict of interest.

## Footnotes

1 http://mtgea.noble.org/v3/

2 http://ljgea.noble.org/v2/
